# Modulatory Effect of Methanol Extract of *Piper guineense* in CCl_4_-Induced Hepatotoxicity in Male Rats

**DOI:** 10.3390/ijerph14090955

**Published:** 2017-08-24

**Authors:** Babatunji Emmanuel Oyinloye, Foluso Oluwagbemiga Osunsanmi, Basiru Olaitan Ajiboye, Oluwafemi Adeleke Ojo, Abidemi Paul Kappo

**Affiliations:** 1 Biotechnology and Structural Biology (BSB) Group, Department of Biochemistry and Microbiology University of Zululand, KwaDlangezwa 3886, South Africa; tunji4reele@yahoo.com (B.E.O.); alafin21@yahoo.com (F.O.O.); 2 Phytomedicine, Biochemical Toxicology and Biotechnology Research Laboratories, Department of Biochemistry, College of Sciences, Afe Babalola University, PMB 5454, Ado-Ekiti 360001, Nigeria; bash1428@yahoo.co.uk (B.O.A.); oluwafemiadeleke08@gmail.com (O.A.O.)

**Keywords:** antioxidant enzymes, carbon tetrachloride, lipid peroxidation, liver toxicity, *Piper guineense*

## Abstract

This study seeks to investigate the possible protective role of the methanol extract of *Piper guineense* seeds against CCl_4_-induced hepatotoxicity in an animal model. Hepatotoxicity was induced by administering oral doses of CCl_4_ (1.2 g/kg bw) three times a week for three weeks. Group 1 (Control) and Group 2 (CCl_4_) were left untreated; *Piper guineense* (PG; 400 mg/kg bw) was administered to Group 3 (T_1_) by oral gavage for 14 days prior to the administration of CCl_4_ and simultaneously with CCl_4_; PG (400 mg/kg bw) was administered simultaneously with CCl_4_ in Group 4 (T_2_); and Livolin forte (20 mg/kg bw) was administered simultaneously with CCl_4_ in Group 5 (T_3_), the standard drug group. The administration of CCl_4_ induces histopathological alteration in the liver, with concomitant increased activities of serum hepatic marker enzymes associated with increased levels of lipid peroxidation. Similarly, there was decrease in non-enzymatic (reduced glutathione) and enzymatic antioxidants (glutathione S-transferase), superoxide dismutase, and catalase. An elevation in serum triglyceride and total cholesterol levels was noticed along with decreased levels of serum total protein. Treatment with PG 400 mg/kg bw exhibited excellent modulatory activity with respect to the different parameters studied by reversing all the above-mentioned biochemical changes significantly in the experimental animals. These results suggest that PG offered protection comparable to that of Livolin forte with better efficacy when pre-treated with 400 mg/kg bw 14 days prior to CCl_4_-exposure.

## 1. Introduction

The current increase in the incidence and prevalence of liver diseases remain a major global health burden. Chronic liver diseases have been identified as the fifth most common cause of death in the United Kingdom, as well as accounting for about 4.8% deaths among American Indians and Alaska Natives [1,2]. The liver plays an essential role in metabolism and the excretion of various therapeutic agents and xenobiotics from the body [3,4]. There has been no definite therapeutic agent for a total cure of liver diseases; documented scientific evidence suggests that most of the available therapeutic agents facilitate the healing or regeneration of the liver [5]. The liver itself has an exceptional regenerative capacity after any cellular injury as a result of xenobiotic exposure (toxic chemicals, ethanol, pesticides, drugs, CCl_4_ etc.). To ascertain the hepatoprotective effect of drugs/molecules during drug screening, Carbon tetrachloride (CCl_4_) induced hepatic injury remains an excellent commonly used model [3].

Exposure to xenobiotics, especially CCl_4_, has been implicated in the etiology and progression of oxidative stress and inflammation in a number of acute and chronic disorders. Direct exposure to CCl_4_ triggers cascades of events. CCl_4_ elicits hepatotoxicity through its biotransformation to trichloromethyl radicals (CCl_3_) or trichloroperoxyl radicals (CCl_3_O_2_), produced by the mixed-function cytochrome P450 oxygenase system of the endoplasmic reticulum, which induces lipid peroxidation of membranes that leads to hepatocellular damage [6,7]. Antioxidant therapies have been shown to play a crucial role in maintaining balance and preventing the body from several diseases caused by the overproduction of free radicals. Naturally, antioxidant therapies act as free radical scavengers, thereby inhibiting lipid peroxidation [8].

Medicinal plants are believed to be a rich source of antioxidants. Added to this, they are a cost effective and potent alternative with few and transient side effects in the treatment and management of liver disorders, when compared with the existing conventional therapeutic drugs available in the market [9,10,11,12]. *Piper guineense* Schum. and Thom. (Piperaceae), widely consumed as a spice in West Africa (commonly called African black pepper; with indigenous names such as Iyere in Yoruba and Uziza in Ibo), contain various biological and pharmacological properties. These properties have therapeutic potential in the prevention and management of hepatotoxicity. *P. guineense* has been recently reported to contain free radical scavengers such as polyphenols, alkaloids, flavonoids, saponins, tannins, and glycosides in appreciable quantities [13]. Previous studies have also reported on the anti-parasitic, antifungal, anticonvulsant, antimicrobial, anti-inflammation activities, as well as the anti-schistosomal and hepatoprotective activities, of *Piper guineense* [13,14,15]. In view of this, the present study was designed to assess the possible protective mechanism of *Piper guineense* against CCl_4_-induced hepatotoxicity and its potential role in the inhibition of oxidative stress in an animal model.

## 2. Materials and Methods

### 2.1. Chemicals

All the chemicals and reagents (analytical grade and the purest quality available) were obtained from Sigma Chemical (St. Louis, MO, USA) and Merck (Zug, Germany), while all kits were Randox assay kits.

### 2.2. Plant Material and Extract Preparation

Fresh *Piper guineense* seeds were obtained from a local vendor in Ibadan, Nigeria. The identification and authentication (voucher No. FHI 107745) were previously carried out by Nwozo and colleagues, 2012 [13]. One kilogram of the seeds was coarsely powdered using an electric blender and macerated with 80% methanol (200 g/1000 mL) with intermittent stirring for 72 h. The methanol extract was filtered, and the filtrate was concentrated in a rotary evaporator to obtain a (brown) crude extract, which was stored in a clean sample bottle at 4 °C. The yield of the extract was 132.74 g (13.27% *w/w*) of the starting material. The portions of the extract used for this experiment were weighed and reconstituted in distilled water daily, just before administration to the animals.

### 2.3. Animals and Experimental Design

Thirty male Wistar albino rats (weighing 180–200 g) obtained from the Animal House of the Department of Biochemistry, College of Sciences, Afe Babalola University, were randomly divided into five groups and classified into control (negative control), CCl_4_ (positive control), T_1_ (pre-treatment), T_2_ (post-treatment), and T_3_ (standard drug). The animals were acclimatized for fourteen days before commencing the experiment and were maintained on standard feed and water *ad libitum.* The animals were kept in well-ventilated cages at room temperature (28–30 °C) and under controlled light cycles (12 h light/12 h dark). All procedures were carried out in compliance with the protocols approved by the Animal Ethical Committee of Afe Babalola University (ABUAD-SCIREC04/14/02/069).

Group 1 (Control) served as the control. Group 2 (CCl_4_) served as the CCl_4_ control. Group 3 (T_1_) received CCl_4_ and *Piper guineense* extract (400 mg/kg) for 14 days prior to the administration of CCl_4_ and simultaneously with CCl_4_ administration. Group 4 (T_2_) received CCl_4_ and *Piper guineense* extract simultaneously (400 mg/kg), while Group 5 (T_3_), the standard drug group, received CCl_4_ and Livolin forte (20 mg/kg bw) simultaneously. The *Piper guineense* extracts (400 mg/kg bw) and Livolin forte (20 mg/kg bw) were given by oral gavage [16]. CCl_4_ (1.2 g/kg bw) was administered orally three times a week (Mondays, Wednesdays, and Fridays) for three weeks to induce toxicity.

### 2.4. Blood and Tissue Collection

The experiment lasted for 21 days, and, on day 22, the overnight fasted animals were sacrificed. Blood samples were collected and allowed to coagulate at room temperature. The clear, non-haemolysed supernatant sera were quickly removed and stored at −20 °C for subsequent analysis. Liver samples were rapidly expunged, weighed, and washed in ice-cold 1.15% KCl solution; they were homogenized in 56 mM Tris-HCl buffer (pH 7.4), comprised of 1.15% KCl, and then centrifuged at 10,000× *g* for 15 min. The supernatant was carefully separated and kept till required for analysis. A histological assessment of the liver was carried out under a light microscope. Small liver samples were fixed in 10% normal saline and then dehydrated and paraffin-embedded for histological assessment.

### 2.5. Biochemical Assays

The ferric reducing antioxidant potential (FRAP) and oxygen radical absorption capacity (ORAC) activities of the methanol extract was determined using the methods described by Benzie and Strain, 1996 [17], and Ou and co-workers, 2001 [18], respectively. Briefly, 10 μL of the diluted plant extracts was mixed with 300 μL FRAP reagent in a 96-well clear plate. The FRAP reagent was a mixture (10:1:1, *v*/*v*/*v*) of acetate buffer (300 mM, pH 3.6), tripyridyl triazine (TPTZ) (10 mM in 40 mM HCl), and FeCl_3_⋅6H_2_O (20 mM). After incubation at room temperature for 30 min, the plate was read at a wavelength of 593 nm in a Multiskan Spectrum plate reader (51118650, Thermo Fisher Scientific, Waltham, MA, USA). Ascorbic acid (AA) was used as the standard, and the results were expressed as μmol AAE/g sample, while the ORAC assay was carried out using a 96-well microplate using a Fluorescence plate reader (51118650, Thermo Fisher Scientific, Waltham, MA, USA). The reaction consisted of 12 μL of diluted aqueous plant extracts and 138 μL of fluorescein (14 μM), which was used as a target for free radical attack. The reaction was initiated by the addition of 50 μL 2,2’-azobis (2-amidinopropane) dihydrochloride (AAPH), and the fluorescence was (emission 538 nm, excitation 485 nm) recorded every 1 min for 2 h. Trolox was used as the standard, and the results were expressed as μmol/g sample. All determinations were done in triplicates. The activities of serum aspartate aminotransferase (AST), alanine aminotransferase (ALT), and alkaline phosphatase (ALP) were determined according to the method of Reitman and Frankel, 1957 [19]. The serum total proteins concentration was assessed using the method described by Henry, 1964 [20]; total cholesterol and triglycerides were evaluated by routine enzymatic methods using Randox commercial kits.

The extent of lipid peroxidation was measured by the malondialdehyde (MDA) content. This was assessed using the thiobarbituric acid method described by Varshney and Kale, 1990 [21]. The levels of reduced glutathione (GSH) in the liver homogenate were estimated using the method described by Beutler et al., 1963 [22]. Glutathione-S-transferase (GST) activity was determined according to Habig et al., 1974 [23]. The level of superoxide dismutase (SOD) activity was determined by the method of Misra and Fridovich, 1972 [24]. Catalase (CAT) activity was determined by adopting the method described by Sinha, 1972 [25].

### 2.6. Statistical Analysis

SPSS version 10.0 (SPSS Inc., Chicago, IL, USA) was used for statistical analysis. The values were presented as the means ± SD of different groups. The data were analysed with one-way analysis of variance (ANOVA). The results were considered statistically significant when *p* < 0.05.

## 3. Results

### 3.1. Antioxidant Capacity of Piper guineense Methanol Extract

Before embarking on the animal experiment, the FRAP and ORAC activities of the methanol extract were determined. The FRAP and ORAC (693.01 ± 0.28 μmol AAE/mL and 207.41 ± 0.16 μmol TE/mL) values obtained compare well with the values documented in the literature (Table 1).

### 3.2. Effect of Piper guineense on Indices of Hepatotoxicity

The current work showed that CCl_4_ treatment markedly increased the activities of liver serum biomarker (AST, ALT, and ALP) enzymes (Table 2). The concomitant administration of PG extract with CCl_4_ showed significant restoration in serum biomarker activities.

### 3.3. Influence of Piper guineense on Serum Total Protein, Total Cholesterol and Triglyceride Levels

The serum level of total protein was remarkably reduced, whereas the total cholesterol and triglyceride levels were elevated in the in CCl_4_-treated rats compared to the control group (Table 3). Treatment with PG extract attenuated the deleterious effect of CCl_4_ on serum total protein, total cholesterol, and triglyceride.

### 3.4. Effect of Piper guineense on Assessment of Oxidative Stress

Summarized in Table 4 is the modulatory effect of PG extract on oxidative stress in CCl_4_-treated rats. The administration of CCl_4_ significantly induced lipid peroxidation and diminished the status of the enzymatic and non-enzymatic antioxidants in the liver. The administration of PG extract adequately inhibited the stimulation of lipid peroxidation and compensated the diminished antioxidant status.

### 3.5. Histological Examination of Rat Livers

**Figure 1:**
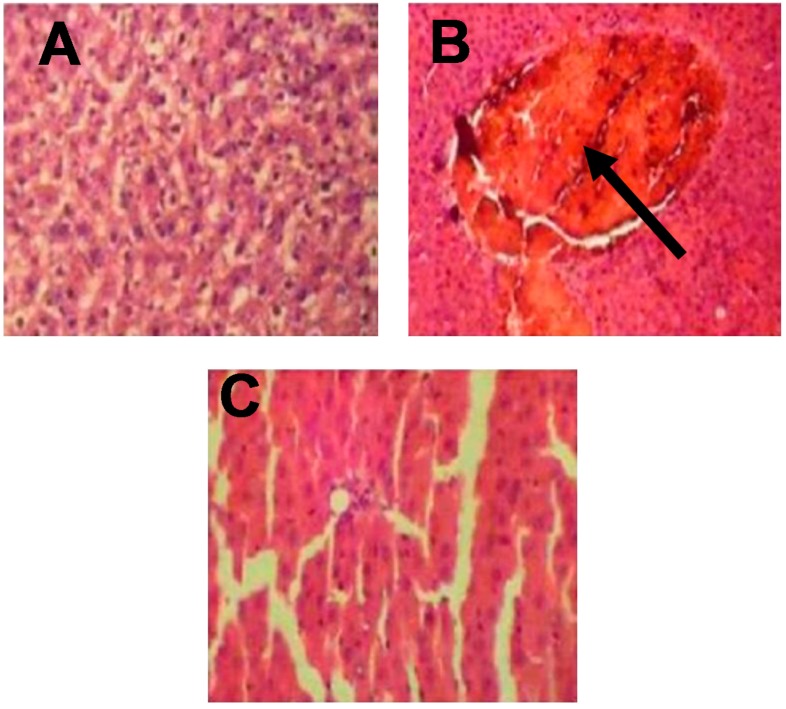

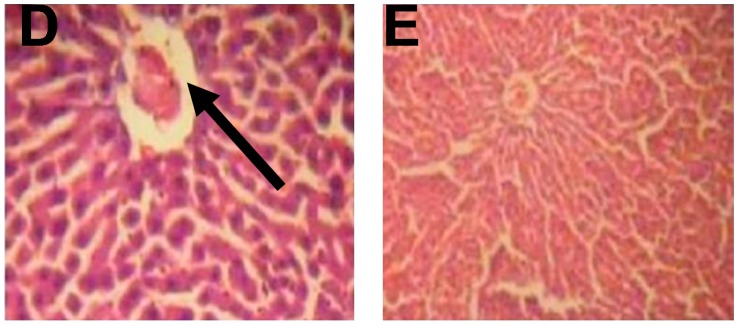
Histological examination of rat livers stained with hematoxylin and eosin (H&E). (**A**) Control: showing normal hepatic architecture with no lesions or abnormalities; (**B**) CCl_4_: showing congestion in the central vein associated with the infiltration of inflammatory cells; (**C**) T_1_: showing mild hepatocytes necrosis and mononuclear cellular infiltration; (**D**) T_2_: showing mild portal tract and lobular chronic inflammation with focal hepatocyte destruction; (**E**) T_3_: showing that improved hepatic architecture, inflammatory cell infiltration, and hepatocytes necrosis were hardly detected (×400).

The results obtained from the histological studies of the liver tissue showing histopathological alterations are presented in Figure 1. The control (negative control) group showed normal hepatic architecture with no lesions or abnormalities, while the CCl_4_ (positive control) group showed congestion in the central vein associated with the infiltration of inflammatory cells. In the T_1_ (pre-treatment) group, mild hepatocytes necrosis and mononuclear cellular infiltration was observed, while, in the T_2_ (post-treatment) group, mild portal tract and lobular chronic inflammation with focal hepatocyte destruction were seen. Inflammatory cell infiltration and hepatocytes necrosis were hardly detected in the T_3_ (standard drug) group.

## 4. Discussion

There has been increased focus on the chemopreventive potential and possible mechanisms of action of plant derived foods in the form of nutraceuticals in the prevention, treatment, and management of liver diseases caused by oxidative stress due to indiscriminate exposure to xenobiotics in our environment. Various studies have shown that the formation of reactive intermediates (CCl_3_ or CCl_3_O_2_ radicals) as a result of CCl_4_-intoxication plays an essential role in the emergence of heaptocellular injury [26]. Before embarking on the animal experiment, the FRAP and ORAC activities of the methanol extract were determined. It is interesting to note that the values obtained are relatively high and compare well with the values documented in literature; this may be an indication that the plant extracts possess antioxidant potential. The higher the absorbance value of the extract, the higher is its antioxidant capacity [27,28].

The results from a pilot study conducted at Afe Babalola University (unpublished data) revealed that the methanol extract was more effective than the aqueous and hexane extracts at the examined dose (400 mg/kg bw), with no significant sign of toxicity. In the present study, the exposure of animals to CCl_4_-treatment resulted in a remarkable increase in the activities of liver biomarker enzymes; notably, AST, ALT, and ALP. Clinically, this suggests that there is a certain degree of damage to the liver. CCl_4_-toxicity has been characterized by a loss of cell membrane integrity, increased permeability of the hepatocytes membrane, and cellular leakage [29,30,31]. Treatment with PG extract (400 mg/kg) exhibited significant restoration of serum markers in the pre-treatment and post-treatment groups when compared with the standard drug group (Livolin forte; 20 mg/kg bw), indicating its protection against CCl_4_-induced hepatocellular injury.

In the present study, it is obvious that the reduction witnessed in the level of serum total protein in CCl_4_-treated rats can be attributed to the initial damage in hepatocytes, causing reduction in mixed function oxidases activity and inhibition of protein metabolism in the liver. The elevation in the serum total cholesterol and triglyceride levels in CCl_4_-treated rats may have resulted from the increase in oxidative stress, which enhances deterioration in hepatic function and the accumulation of lipids, leading to a fatty liver due to the failure of their secretory mechanisms [32,33,34,35]). Treatment with PG extract (pre-treatment and post-treatment) attenuated the deleterious effect of CCl_4_ on serum total protein, total cholesterol, and triglyceride and caused a subsequent recovery towards normalization.

The present increase in lipid peroxidation and the consequent suppression of antioxidant activities in CCl_4_-treated rats has been well documented in the literature as an indication of oxidative stress [36,37,38]. Free radical mediated lipid peroxidation via the formation of reactive intermediate (CCl_3_ or CCl_3_O_2_ radicals) is the major mechanism of hepatocellular injury by CCl_4_ [36,39]. The cell protects itself from oxidative damage by recruiting reduced glutathione (GSH) and scavenging enzymes such as CAT, SOD, glutathione peroxidase (GPx), and GST as first-line cellular defenses in response to oxidative challenges in order to protect cellular integrity. Suppression in the status of these enzymatic and non-enzymatic antioxidants in the hepatic homogenates is an indication of the overwhelming effect of CCl_4_ on their normal redox state within the cells [40,41,42,43].

Accordingly, the oral administration of PG extract significantly repressed the CCl_4_-induced elevation of lipid peroxidation and also enhanced the status of these enzymatic and non-enzymatic antioxidants. This complemented the previously identified protective roles (the ability to restore hepatocytes and protect membrane integrity, as well as the ability to reduce the leakage of the hepato-specific enzymes) of PG extracts [13]. The mode of action of *P. guineense* extracts in this experiment is similar to that of Livolin forte [44]. The histological examination of the liver samples strongly supports the modulatory role of *P. guineense* extract in CCl_4_-induced toxicity.

## 5. Conclusions

The results obtained from this study demonstrate that extracts from *P. guineense* possess antioxidant and hepatoprotective properties comparable to that of Livolin forte, with better efficacy when pre-treated with 400 mg/kg bw 14 days prior to CCl_4_-exposure in an animal model. We propose that the possible mechanism by which PG brought about the observed changes in the present study may be due to the synergistic interactions of its bioactive components. Therefore, as we investigate nature’s recipes for health, *P. guineense* is a candidate with promising therapeutic potential.

## Figures and Tables

**Table 1:** The antioxidant capacity of *Piper guineense* methanol extract.

Parameter	Value in *Piper guineense*
Ferric reducing antioxidant potential (FRAP) (μmol AAE/mL)	693.01 ± 0.28
Oxygen radical absorption capacity (ORAC) (μmol TE/mL)	207.41 ± 0.16

**Table 2:** The effect of *Piper guineense* on indices of hepatotoxicity.

Groups	Aspartate Aminotransferase (AST) Activity (U/L)	Alanine Aminotransferase (ALT) Activity (U/L)	Alkaline Phosphatase (ALP) Activity (U/L)
Control	34.84 ± 1.12 ^b^	21.35 ± 0.18 ^b^	109.87 ± 0.43 ^a^
CCl_4_	56.12 ± 1.08 ^a^	29.09 ±0.12 ^a^	145.88 ± 0.38 ^b^
T_1_	42.55 ± 1.51 ^a,b^	21.87 ± 0.13	117.72 ± 0.08
T_2_	51.96 ± 1.36 ^a^	23.90 ± 0.71	138.09 ± 0.18 ^a^
T_3_	44.70 ± 1.34 ^a,b^	23.34 ± 0.16	119.09 ± 0.51

The values shown are mean ± S.D. (*n* = 6). Mean differences are significant (*p* < 0.05) when compared with: ^a^ control group, ^b^ CCl_4_ only.

**Table 3:** Effect of *Piper guineense* on serum total protein, total cholesterol, and triglyceride levels.

Groups	Total Protein (g/dL)	Total Cholesterol (mg/dL)	Triglycerides (mg/dL)
Control	29.26 ± 0.23 ^b^	142.44 ± 0.26 ^b^	46.64 ± 0.14 ^a^
CCl_4_	23.49 ± 0.36 ^a^	261.22 ± 0.20 ^a^	98.42 ± 0.82 ^b^
T_1_	34.36 ± 0.36 ^a,b^	189.66 ± 0.02 ^a,b^	49.19 ± 0.11
T_2_	27.51 ± 0.14	223.03 ± 0.16 ^a,b^	63.98 ± 0.28 ^a^
T_3_	32.77 ± 0.27	201.86 ± 0.23 ^a,b^	51.21 ± 0.24 ^a,b^

The values shown are mean ± S.D. (*n* = 6). Mean differences are significant (*p* < 0.05) when compared with: ^a^ control group, ^b^ CCl_4_ only.

**Table 4:** The effect of *Piper guineense* on the assessment of oxidative stress.

Groups	Lipid Peroxidation (LPO)	Reduced Glutathione (GSH)	Glutathione-S-Transferase (GST)	Superoxide Dismutase (SOD)	Catalase (CAT)
Control	13.35 ± 1.45 ^b^	41.21 ± 0.41 ^b^	19.12 ± 1.89 ^b^	7.09 ± 0.11 ^b^	24.21 ± 0.71 ^b^
CCl_4_	28.40 ± 1.82 ^a^	28.26 ± 0.26 ^a^	12.61 ± 1.08 ^a^	3.41 ± 0.26 ^a^	16.79 ± 0.86 ^a^
T_1_	19.14 ± 0.89 ^a^	37.56 ± 0.43 ^b^	18.86 ± 1.13 ^b^	7.23 ± 0.16 ^b^	23.61 ± 0.53 ^b^
T_2_	23.92 ± 1.14 ^a^	35.05 ± 0.33 ^b^	15.08 ± 1.79	5.91 ± 0.13	19.20 ± 0.29
T_3_	17.69 ± 1.86 ^a^	37.34 ± 0.21 ^b^	19.59 ± 1.46 ^b^	7.01 ± 0.08 ^b^	21.44 ± 0.12 ^b^

The values shown are mean ± S.D. (*n* = 6). Mean differences are significant (*p* < 0.05) when compared with: ^a^ control group, ^b^ CCl_4_ only. Units: LPO (unit/mg protein), GSH (µg/mL), GST (µm/min/mg protein), SOD (units/mg protein), CAT (units/mg protein).
